# Cleanliness

**Published:** 1879-09

**Authors:** 


### CLEANLINESS.

In 1872 several cases of typhoid fever occurred in families living in Leeds, England. These families were dotted about in different localities without any apparent regularity. On close examination the physician found that every family attacked was supplied with milk by the same man, but his family had uninterrupted health. They used none of the milk themselves. He obtained his milk from a farm house in the country. On visiting the farm it was ascertained that there were six persons in the family lying ill with typhoid fever, in a room adjoining the one in which the milk cans were kept, and that the woman who handled the milk also nursed the sick. During the summer of 1873 a dozen or more inmates of a boarding school were taken ill, showing typhoid symptoms. On examining into the milk supply, it was ascertained that the water used to wash the milk vessels, came from a well into which was leaking a water-closet pipe.

